# Cancer Education in Poland: Current Status and Suggestions for Improvement

**DOI:** 10.1007/s13187-016-1033-2

**Published:** 2016-04-15

**Authors:** Joanna Wawryka, Paulina Ziobro, Maciej Tyszko

**Affiliations:** Faculty of Medicine, Wrocław Medical University, J. Mikulicza-Radeckiego 5, 50-345 Wrocław, Poland

## Abstract

In this article, we want to describe the opportunities we experienced though involvement with the AACE and EACE to improve cancer education in poland from our points of view as fifth-year medicine students. By participating in the annual meeting of the EACE that our university (Wroclaw ) hosted and also the ESMI-ESO course on medical oncology for medical students, we were able to improve our doctor- patient skills and deepen our knowledge caring for cancer patients. In our opinion in the obligatory medical curriculum in Poland, the curricular offerings in oncology should be better coordinated and there is too little attention to the teaching of the so called “soft skills” for future doctors. Over the course our studies, we are taught a great deal about the biology, diagnosis and symptomatology of cancer; however, we are not exposed very much to issues of communication between the doctor and the oncology patient, or appropriate strategies to pass information about the diagnosis and prognosis. Therefore, we feel that it is important for the future for students to learn more about such topics. Many do this in informal, extracurricular ways as there is much interest to learn about such topics and we will discuss several useful tools. In this review, we would like to summarize the current state of oncology education in Poland and our hopes for improving the current state and to emphasize how inspiring it was for us to participate in an international cancer education meeting where we could discuss good ideas from all over the world and bring them home to Poland.

We, the authors, are fifth-year medical students at the Wroclaw Medical University. In just one year, we will complete our undergraduate studies and begin to work at a hospital. We were inspired to write this paper through our participation in the 2013 European Association for Cancer Education (EACE) hosted by our university and we left that meeting, having discussed exciting ideas with colleagues from many countries. We are dedicated to make improvements in the Polish educational system about the approach to cancer. Taking into consideration the knowledge that we have acquired during our studies, we would like to discuss the current status of cancer in Poland, and oncology education in Poland for both medical students and doctors at the beginning of their careers. In the course of our own education, we have experienced some reforms of the curriculum which we will describe and we will give our opinions about future possible ways to improve oncology (education) awareness in our country.

We will first examine the state of cancer as a health problem in Poland. We will discuss the epidemiology of cancer, assess how serious a problem cancer is for our country, and evaluate the actions taken and their influence on outcomes. In Poland, like other developed countries, cancer morbidity is an important problem that is increasing among both men and women. In 2012, 152,000 poles were diagnosed with cancer (Poland has a population of around 38 million). The most common cancers in men are lung, prostate, and colorectal cancer while in women, the most common are breast, colorectal, and lung cancer [1]. While the mean morbidity rate in Poland is lower than that in other European Union countries, the mortality is, in our opinion, still too high, and there is a steady increase in the number of new cases of cancer in Poland. This increase is explained by population growth, changes in age structure of the population with an increase in the population of age groups in which cancer morbidity is particularly high, the persistence of unhealthy population behaviors (smoking, alcohol, unhealthy diet) and last, but not least, an unsatisfactory improvement of the early diagnosis.

The national cancer registry at the oncology center in Warsaw is responsible for the compilation of statistics of cancer morbidity and mortality. Since 1952, each new case of cancer must be reported from both public and non-public treatment centers. Healthcare centers, and all medical practitioners must complete a cancer report card with each new case diagnosed and the collected data is then sent monthly to 1 of 16 voivodeship cancer registries. The data about mortality is compiled by the register office and the central statistical office from death certificates. The compiled data is published in information bulletins. The good news is that statistics published by the national cancer registry, Marie-Curie Institute, show that the number of deaths caused by cancer is decreasing, yet it is still not satisfactory [2, 3].

The newest generation of young doctors at the beginning of their careers is the best hope for improvement of the standards for oncology treatment. However, the level of education is highly variable, and this variability is the major reason we wrote this paper.

We feel that there are readily available solutions, especially after our interactions with cancer education experts from all over the world.

We wish to next introduce the general structure of medical studies in Poland. At a medicine faculty, the course of medicine which took 6 years in the past was changed to 5 years in 2012. In a 6-year course in the past, the first 3 years consisted of pre-clinical courses and the last 3 years were clinical. In the newer 5-year course, the pre-clinical part has been reduced to 2 years. Classes such as biology, biochemistry, histology, cytophysiology, genetics, pathomorphology and pathophysiology allow each student to learn the characteristics of a cancer as well as hypotheses about the origins, but these courses are often not coordinated so a comprehensive picture of cancer may not be introduced. Concrete theoretical knowledge is required to begin the clinical part of the curriculum. Recently, some of the universities in Poland (e.g., Wroclaw) have introduced a separate course in the pre-clinical part called propaedeutics of oncology which gives a more coordinated and comprehensive look at cancer. At Wroclaw, there are twenty hours of seminars about the epidemiology, symptoms, prophylaxis, and treatment strategies for cancer. This course takes place at the third year of the medical studies and one of our suggestions is to have each university take such a coordinated, comprehensive approach.

In the clinical years, there is also a course on oncology (at the sixth year in Wroclaw). During 10 hours of lectures, students deepen their theoretical knowledge but they also experience 50 hours of clinical training which allows them to meet oncology patients. This is a wonderful way to experience applied knowledge in real situations. Our second suggestion is for all universities to develop such experiential clinical experiences and for students to see role modeling and decision making by the various oncology specialists. Students are taught different treatment strategies and learn about the obstacles that occur in treatment. This capstone course concludes with a verification of the achieved knowledge level in the form of a final test exam [4]. However, even with these coordinated efforts, the total load of such oncology courses is only 80 hours and this is only 3.35 % of total course load during the studies.

We advocate for more attention to this important set of diseases. Another obligatory course at the last year of our studies is palliative medicine, introducing the principles of the care of terminally ill patients. Naturally, other clinical courses such as internal medicine, surgery, gynecology, dermatology, and pediatrics also cover some aspects of oncology; nevertheless, the exposure to cancer topics for polish students is haphazard and students may not be exposed to the current medical standards in oncology.

One important development began in 2003 when the first Poland Independent Laboratory of Oncology Education at the Medical University of Warsaw was opened. This program deals with education about epidemiology, prophylaxis and the treatment of cancer as well as collects statistics concerning morbidity and treatment outcomes. Currently, similar centers operate at other provincial oncology facilities. However, according to data collected by W. Chmielarczyk, only 9 out of 21 cancer centers have an oncology education office [4]. This suggests that oncology education is still underestimated in Poland and there is insufficient attention put to propagating organized and systematic oncology education. Lack of organizational and financial stability, may be the root of the problem. Undoubtedly, we should seek to promote the creation of effective cancer education offices at each cancer center and medical faculty as a way of spreading oncology knowledge.

After medical school, each student does internship for a year. Polish medical students from the beginning of their careers are aware of the gravity of cancer. Unfortunately, during the earliest years of medical education in Poland, too little focus is put on developing soft skills, e.g., conversation with oncology patients or providing information about the diagnosis and prognosis. We often forget that oncology is about the patients with cancer, and not just about malicious cells [5, 6]. Unfortunately, in the course of studies we fear that our universities do not promote the holistic approach; therefore, it needs to be acquired by the students on their own. Many young doctors, agree with the theories of humanistic oncology promoted by prof. Tadeusz Koszarowski. Students see the need to create a separate coordinated approach in the future which could better set up cancer therapy strategies in practice [7, 8]. Compared to foreign centers, in the USA, for example, where narrative oncology classes are a part of medical studies, in Poland, such a subject is almost neglected [9]. The introduction of such activities in Polish medical universities would very likely help to improve the communication between doctor and oncology patient and thus provide a better understanding of the plight of the patient. Thus, at most schools and during internship, there is no course focused on the principles of doctor–patient communication; such knowledge has to be achieved by the students by observation of good and not so good role models and mostly on their own, often extracurricularly and the method is usually trial and error. This results in many cases, with the situation that a young doctor may be unable to communicate well with a patient and we feel this lack of instruction about communication is the biggest place for improvement in the polish system. It is our strong opinion, that the approach towards skills such as communication and ways to change unhealthy behavior would be important ways to improve cancer education and thus, impact future cancer outcomes.

We are proud of our medical education and we must report that the education not only happens in Poland but also in other countries, and not only in the classroom and clinic, but in student initiated programs that dig deeper into special interests students have. The subject of oncology has created much special interest. In Poland, medical education is carried out at a high level, and this attracts foreign students from countries like Denmark, Sweden, Norway, and Germany. Furthermore, Polish students eagerly participate in exchange programs—both to study and to practice in other European countries. Trainings and internships concerning oncology, along with courses organized both in Poland and abroad, are very popular among students—e.g., ESMO-ESO course on medical oncology for medical students. Such exchanges are beneficial to each country involved when we can share best practices.

There is a great hunger for and passion for studying cancer in polish students as evidenced by the great interest through studying oncology extracurricularly. The Medical University in Wroclaw runs a scientific society, which unites scientific clubs from all medicine areas, where students can develop their abilities and interests. Among the 158 active scientific circles, 11 concern cancer. Each of them attracts many students and includes subjects like hematology, pediatric hematology, surgical oncology, pathomorphology, cytology, medical oncology, urologic oncology. Still, even with all these opportunities, there is more interest in learning about oncology—for example, students organize numerous conferences on oncology topics for fellow students, and invite specialists to share their knowledge and experience with these future doctors. The subjects of these meetings are diverse, from general issues like “Man versus cancer” and “woman versus cancer” to very specific fields like neuroendocrine tumors or becoming a bone marrow donor. The attendance is always very high and the meetings receive enthusiastic reviews by students.

In Poland, medical volunteer programs are also very popular, and through them, future doctors can expand their skills, verify acquired knowledge in practice, and look at the work of experienced specialists. In addition, academic mentors encourage their students to collect materials and to write articles for scientific journals.

As a result of this, students can acquire the most up-to-date knowledge in a particular field and also can learn the basics of statistics and the proper form of editing scientific papers. The students also travel to learn more about modern treatment and diagnostic techniques by attending various congresses and workshops in different countries all over the world.

Each year, the students at our medical university in Wroclaw take active part in the annual meeting of European association of cancer education (as we did) and ESMO-ESO course on medical oncology for medical students. There, papers concerning different subjects ranging from palliative care to doctor–patient communication are presented. The students listen to and learn from experienced specialists as they deepen their knowledge and create ideas for change, just as we did.

Important new ideas are also raised by the American Association for Cancer Education and the European Association for Cancer Education and these are enthusiastically received by the students. The benefits are twofold. The conscience of how important oncology education is starts during the studies, while the cooperation with the doctors from foreign medical centers opens up various possibilities for the future.

After internship, young doctors decide on specialization. Currently in Poland, (Table 1) there are a total of 2917 professionals in oncology related fields, 726 medical oncologists, 713 radiotherapists, 707 oncology surgeons, 168 pediatric oncologists, 204 gynecologic oncologists and 399 hematologists. This number is insufficient for the needs of the growing number of patients [10]. This lack of sufficient specialists was recognized, and in 2014, the number of residencies for the education of future oncologists was significantly increased (to 352—which is still insufficient).

**Table 1:** Oncology specialists in Poland (February 2015)

Specialization	Number
Clinical oncology	726
Radiotherapy	713
Oncology surgery	707
Children oncology	168
Oncology gynecology	204
Hematology	399
Total	2917

In fact, in order to solve the financial and technological problems of providing cancer care, international cooperation of scientific centers is crucial. Polish doctoral students eagerly carry out projects in cooperation with many international universities and cancer research centers. This type of basic research is important to push forward the state of the art in cancer medicine [11, 12].

We believe that all doctors need to know about cancer because it touches so many fields. Family doctors play a special role in early diagnosis of cancer as they are the ones the patients will visit in the first place with concerning symptoms. Employees of the primary health care should always be prepared with oncology awareness to be able to send patients presenting suspicious symptoms to oncology specialists as soon as possible for a diagnosis verification, and when necessary, to be able to diagnose a e cancer at a stage when it is more likely to be curable and have a more positive outcome. Another duty of family doctors is to take care of the patients after and sometimes during oncology treatment. In addition, a medical doctor in the polish society is usually seen as an educator; therefore, they are the ones who should promote healthy behaviors in order to prevent chronic diseases including cancer. A family doctor is usually the healthcare professional closest to the patient and should have the best chance of getting him /her to understand a message of health promotion [13].

Because of the above, it seems obvious that family doctors should be the target of an educational offerings about oncology and to always be improving their knowledge about the symptoms, diagnosis and therapy of cancer, as well as ways to minimize the side effects of the cancer treatment. This knowledge should be kept up-to-date continually as this is probably the best way to improve the success against cancer.

However, in Poland, we found that there is much need for improvement in oncology education for family physicians. W. Chmielarczyk, with his associates, studied the oncology knowledge of the family doctors, primary healthcare doctors and nurses—the participants of a 1-day course about the diagnosis of cancer and post-therapy care [13]. They used the results of questionnaires completed by 660 doctors and 330 nurses participating in 1-day oncology diagnosis courses. The questionnaire was anonymous, filled in by the participants themselves after the training. To assess the results, 100 questionnaires were chosen at random.

The results are quite disturbing. Both the nurses and the doctors showed an alarmingly low level of oncology education. Even though most of them declared that at their centers and workplaces the principles of oncology education are being followed, the results proved the knowledge about the principles insufficient. As a conclusion, another recommendation we have is to focus on developing effective ways to educate this work group [13].

There is effort though. Organizations such as the college of family physicians in Poland, the polish society of family medicine and the polish union of oncology organize trainings, conferences and courses all over Poland, where family doctors have the opportunity to update their knowledge. Moreover, a journal family medicine forum is being published, dealing with important and practical problems in an educational way.

There is also a website for family physician’s academy (www.alr.edu.pl), where training videos about the most common diseases are available. A special helpline operated by an experienced oncology specialist was created for the family doctors to help them with a diagnosis or treatment of their patients.

In Poland, the most frequent cancers such as breast cancer, cervical cancer and colorectal cancer are covered with free screening. Women aged 50–69 can receive mammography to diagnose early breast cancer every 2 years. However, according to data published by the national center against cancer, in 2012, mammography screening rate was 46.96 % and in 2013 47.18 %. There is an upwards trend but much room for improvement. There are many educational initiatives to convince polish women to participate in such examinations. Many events, conferences, educational campaigns and workshops, addressed to various social and professional groups, were carried out. What is more, invitations for the examinations are sent to selected women, and this action seems to be most effective [14]. Another screening program for the polish population is the early detection of cervical cancer program, addressed to all women aged 25–59 years, once every 3 years. In 2012, screening rates for cervical smear examination were 35.71 % while in 2013, as much as 44.10 %, which clearly shows the growing interest and increased health awareness in women, which is largely due to numerous educational campaigns but once again, there is much room for improvement. [14]. Unfortunately, the situation is much worse in small towns. According to the observations of W. Chmielarczyk, the attendance in cities with less than 20,000 inhabitants usually does not exceed 22 % and the reason for such a bad result might be a poor organization of information about the actions within communities, as well as in the lack of interest of non-medical opinion-forming units in promoting healthy behaviors [15]. This refers to, for example, announcements in the Roman Catholic parishes, department stores, community centers, and schools. Only in a few places was there any information about the screening. The results make us think on how we can reorganize the information transfer in small towns and how to activate local authorities and non-medical institutions to encourage their residents to take care of their health through participation in screening programs. Another very important screening program carried out in Poland in order to reduce mortality from colorectal cancer is colonoscopy. It is addressed to people over 45 years old and those with a positive family history of a specific cancer. Many organizations are involved in promoting public oncology education in Poland. These include the polish society of clinical oncology, along with the foundation there and back again. These programs educate patients, their families, and medical staff, including young doctors.

We feel they should also be more involved with our medical student education. For this purpose, publishing series like “together we can beat the cancer” and “what to know” were created under the patronage of organizations such as the Ministry of Education, Polish Cancer Society, Polish Society of Surgical Oncology, and Polish Union of Oncology. Medical students and young doctors should be aware of these programs and perhaps can be partners in developing or disseminating materials.

There is much good happening on a governmental level. The actions designed to reduce both cancer morbidity and cancer mortality in Poland are financed by the government, the Ministry of Health. The National Program for Fighting Cancer was created; within the program, PLN 250 million (approx. 62 million euros) are spent on the fight against cancer annually.

According to the new resolution of the Council of Ministers concerning the multiannual program for the years 2016–2024, oncology education was also included. Oncology training in pre- and post-graduate education will be developed and expanded not only for medical doctors but also for dentists, psychologists, nurses, midwives and other medical professionals. This is an important new initiative another important new initiative is that in Poland, since 1 January 2015, the government has implemented a project for fast oncology diagnostics, which, as a collection of legal documents, has been published under the name of the oncology packet.

This is produced under the patronage of the ministry of health and the national healthcare fund. The project aims to make it easier for patients with a cancer suspicion to reach specialists, shorten the queues by abolishing the limits for oncology procedures, and set the diagnostics guidelines.

A family doctor and a primary healthcare doctor will be able to order more procedures for a patient presenting suspicious symptoms in order to formulate the initial diagnosis as soon as possible. A patient suspected of having a cancer receives a special diagnostic and treatment card enabling him to visit a specialist directly. There, a medical doctor should perform adequate, specialized research to confirm or disprove the initial diagnosis within 9 weeks. Afterwards, a joint consultation involving several relevant specialists takes place, the therapy is decided and a personal doctor—supervising coordinator—is appointed as the person responsible for the proper course of the treatment in hospital. Convalescents remain under the constant care of their family doctors. All activities are aimed at reducing mortality and the cost of the treatment by early diagnosis of malignant tumors. [16]. Along with the introduction of the oncology packet, the Ministry of Health has organized trainings about oncology for the family doctors and primary healthcare doctors.

The social learning theory, social cognitive theory, which says that medical students and young doctors should keep in mind that they are a medical authority for the surrounding environment is, therefore, an important aspect [4, 14, 17].

It should also be noted that our young doctors have a chance to develop their skills under the european organization for research and treatment of cancer (EORTC). It is particularly important that the organization grants the possibility to study rare types of cancer and supports young scientists under various scholarship programs. A course organized for 12 consecutive years by ECCO-AACR-EORTC-ESMO is a good example of an opportunity for polish doctors to learn about clinical oncology research. This course, taught by 25 experts from all over the world, is undoubtedly one of the best methodologically designed, intensive trainings for aspiring clinical scientists interested in all oncology areas, dealing with problems of design and principles of clinical research. During a week-long training, the participants prepare a full protocol of a clinical study. In 2010, two representatives from the Center of Oncology Institute (Warsaw, Poland) took part in the films course [17, 18].

Another institution that has been a great resource for polish physicians interested in cancer is the American College of Surgeons, which prepared many educational program aiming to improve the outcomes of the surgical treatment and to increase the quality, e.g., commission on cancer, national accreditation program for breast centers, and quality improvement program. ACOS propagates the newest treatment methods and clinical outcome evaluations from different centers. Some of the projects are designed exclusively for students, e.g., medical student simulation-based surgical skills curriculum, made in cooperation with the association for surgical education.

All of these programs offer tremendous opportunities for polish doctors to learn more about cancer. As the doctors learn more, this will surely trickle down to the oncology education for medical students.

Our suggestions:

1. Coordinate the pre-clinical and clinical courses with a focus on oncology.
2. Improve programs that teach the so called “soft skills” like communication and behavior change strategies.
3. Capture what is being learned by polish doctors attending international course and conferences and doing research at prestigious cancer programs.
4. Capitalize on all the extracurricular student interest in the subject of oncology. There is a passion there that can help to improve outcomes in the future.
5. Increase the number of oncology specialists and encourage young students and young doctors interested in oncology to pursue these opportunities. Connect passionate students with mentors who will help them thrive.
6. Have student like we did attend meetings like aace and eace, read this journal and work together to incorporate ideas they learn to help improve the education and eventually the outcomes for cancer patients in Poland.
7. Make students aware of the governmental programs to speed diagnosis, educate front line primary caregivers, primate screening and early diagnosis and foster behavioral change of unhealthy habits and adoption of healthy ones.

The task of spreading both the theoretical and practical oncology knowledge among medical students begins with of the medical universities. They provide the students with systematic classes, during which the future doctors, in accordance with the accepted standards, get to know—regardless of their future specialization—the basic information about cancer. All doctors need to know about oncology, and those with the passion to pursue specialization should be encouraged.

The issue of medical education does not confine to the university walls during the training of the medical staff; such policy would be bound to fail. To achieve a significant improvement in the results of treatment, a much broader approach is needed, for example carrying out education campaigns in the society, as it is well known that prophylaxis and early cancer diagnosis is the best way to succeed in the fight against cancer. We feel that the biggest challenge is to build a strategy of effective communication between doctors and patients that will be understandable for people lacking extensive medical knowledge and will be persuasive and encouraging to change unhealthy habits and adopt healthy ones.

Oncology education is an important part of the whole culture that surrounds the disease called cancer in a society. Even at the early part of our careers, we have learned that each country has a slightly different strategy for the improvement of the outcomes of the cancer therapy; learning from the experience of others and applying the best practice, will help to achieve success in Poland. The training of young doctors is only one of many steps in a long ladder of oncology culture. Over the years, we have observed increased interest in this area and many reforms of our education system have been enacted and are visible to us even early in our own education. We were inspired by participating actively in the 2013, the annual meeting of the European association of cancer education in our home university. Current important issues of the oncology education were raised. And the dialog was very stimulating. We were included as full partners in the meeting. This experience at EACE allowed us to deepen and bring up-to-date ideas from the experience of multiple countries. Further, our membership in organizations such as the american association of cancer education and the european association of cancer education has opened for us the possibilities for the young doctors to develop their knowledge systematically and implement new solutions in their own healthcare centers the scientific magazines help to spread the oncology education, especially the journal of cancer education, where the latest reports on oncology can be published. Our report in this journal is meant to emphasize how much students can do to change things for the better by becoming participants in these organizations.

Although, as students, we do not yet have an experience in oncology education, with this article we would like to point out what we think are the good and the bad aspects of the oncology education in Poland. The standards of teaching of the young oncology specialists in Poland are high, which gives us hope for improving the detection rate of cancer and treatment outcomes of the oncology patients. The passion for and interest in the subject is even higher. What is more, an integrated education aimed at the society, particularly at the family doctors, oncology patients and their families, seems to be the best way to achieve the goal being a reality in which cancer can become a less serious social problem in the future.

In our opinion, the most important opportunity for improvement of oncology education lies in teaching more about the soft skills, such as the ability to communicate with the patient and to pass the information about the health status and the prognosis. Although, at this moment, those skills are not in the curriculum, we find it important to introduce classes in doctor–patient communication, where a student will be prepared to talk with the patient in a caring and at the same time efficient way. This is important in every field of medicine, not only in oncology. It would surely grant the skills to build trust and to know the patient better, which increases the chances of a successful treatment.

We feel privileged and honored to be encouraged to write this paper as young people beginning our professional lives and inspired to make things better.
